# Improving Care Transitions for Sepsis Survivors to Home Health Care: The I-TRANSFER Experience

**DOI:** 10.1093/geroni/igaf122.4386

**Published:** 2025-12-31

**Authors:** Kathryn Bowles, Sang Bin You, Michael Stawnychy, Nancy Hodgson, Elaine Sang, Katherine Pitcher, Sungho Oh, Karen Hirschman

**Affiliations:** University of Pennsylvania School of Nursing, Philadelphia, Pennsylvania, United States; University of Pennsylvania, Philadelphia, Pennsylvania, United States; University of Pennsylvania School of Nursing, Philadelphia, Pennsylvania, United States; University of Pennsylvania, Philadelphia, Pennsylvania, United States; University of Pennsylvania School of Nursing, Philadelphia, Pennsylvania, United States; University of Pennsylvania School of Nursing, Philadelphia, Pennsylvania, United States; University of Pennsylvania School of Nursing, Philadelphia, Pennsylvania, United States; University of Pennsylvania, Philadelphia, Pennsylvania, United States

## Abstract

Older adult sepsis survivors are at high risk for readmission, with a median time-to readmission of 11 days, making timely attention post-discharge critical. However, providing timely post-acute care is difficult partly due to care transition challenges —such as barriers in identifying sepsis, sharing critical information, and activating timely post-acute follow-up. I-TRANSFER evaluates the implementation and impact of a protocol for timely home health (HH) and outpatient visits within one-week post-discharge for Medicare sepsis survivors. Herein, we describe ERIC implementation strategies used to overcome barriers to timely post-acute care during the I-TRANSFER implementation. Five health systems (16 hospitals) in four states partnered on implementation with five HH agencies. Barriers and facilitators identified via needs assessment interviews were coded using thematic analysis. Findings were shared and strategies developed with implementation teams over 18 months. Strategies were mapped by the research team to the Expert Recommendations for Implementing Change (ERIC) taxonomy. Barriers and strategies were discussed in 36 planning and 145 implementation meetings. To identify sepsis patients, sites modified EHRs (alerts/reports), educated providers, and improved documentation. For information transfer, strategies included referral flags, reminders, notes, staff education, and securing a new sepsis aftercare ICD-10 code. For timely outpatient care, appointments were achieved using centralized support, revised roles, and telemedicine. Implementation strategies addressed barriers from diagnosis through outpatient follow-up, offering actionable guidance and scalable solutions to enhance hospital-to-home and outpatient transitions for older adults and prevent poor discharge outcomes. Mapping to the ERIC taxonomy provides a standardized way to describe the strategies across implementations.

